# Supplementary figures and images for: Sea urchin harvest inside marine protected areas: an opportunity to investigate the effects of exploitation where trophic upgrading is achieved
Source: PeerJ. 2022 Mar 7;10:e12971. doi: 10.7717/peerj.12971 (PMC8908888; doi:10.7717/peerj.12971)

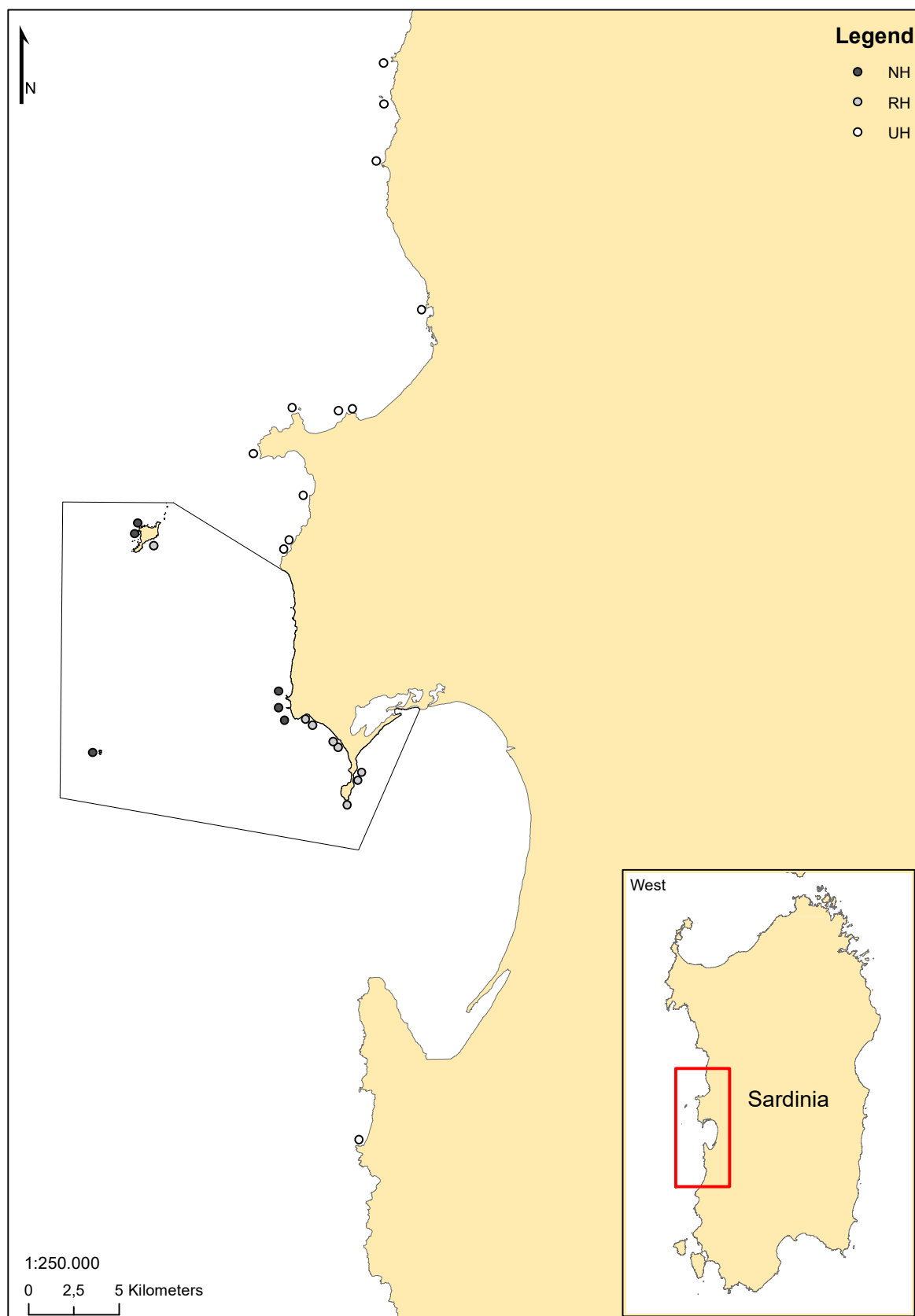

Fig. S1

Supplement: Supplemental Information 1 [file peerj-10-12971-s001.pdf]

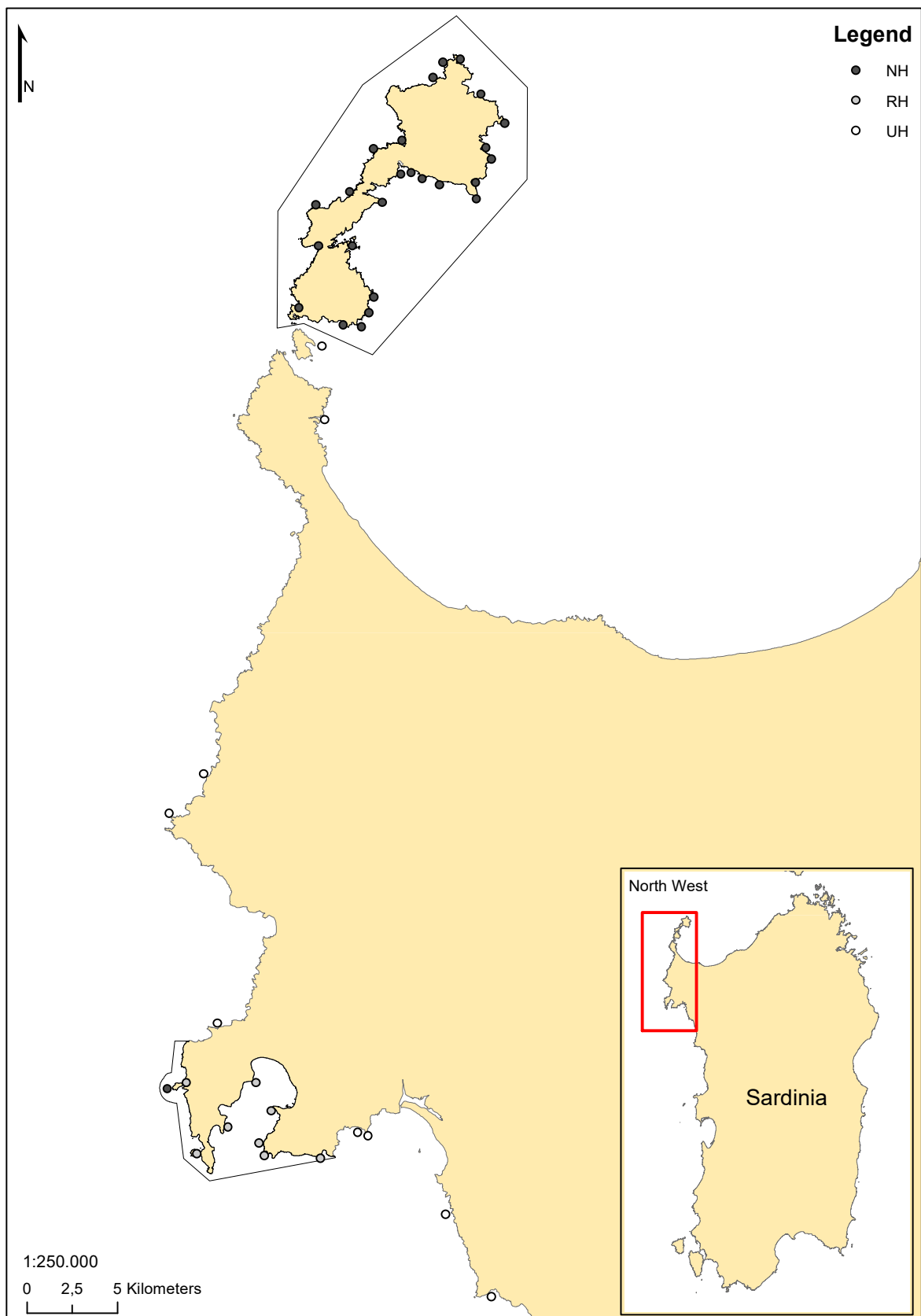

Fig. S2

Supplement: Supplemental Information 2 [file peerj-10-12971-s002.pdf]

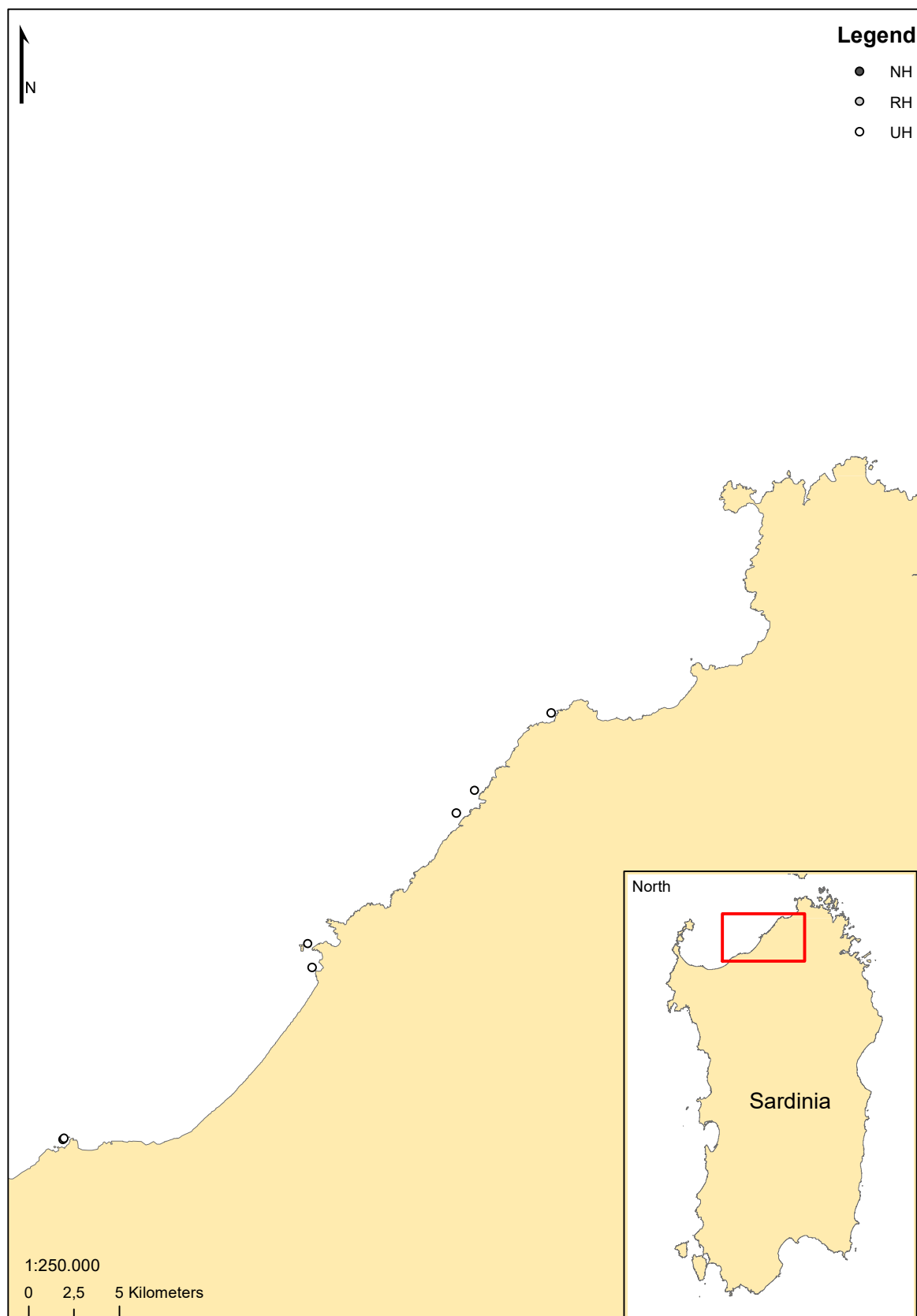

Fig. S3

Supplement: Supplemental Information 3 [file peerj-10-12971-s003.pdf]

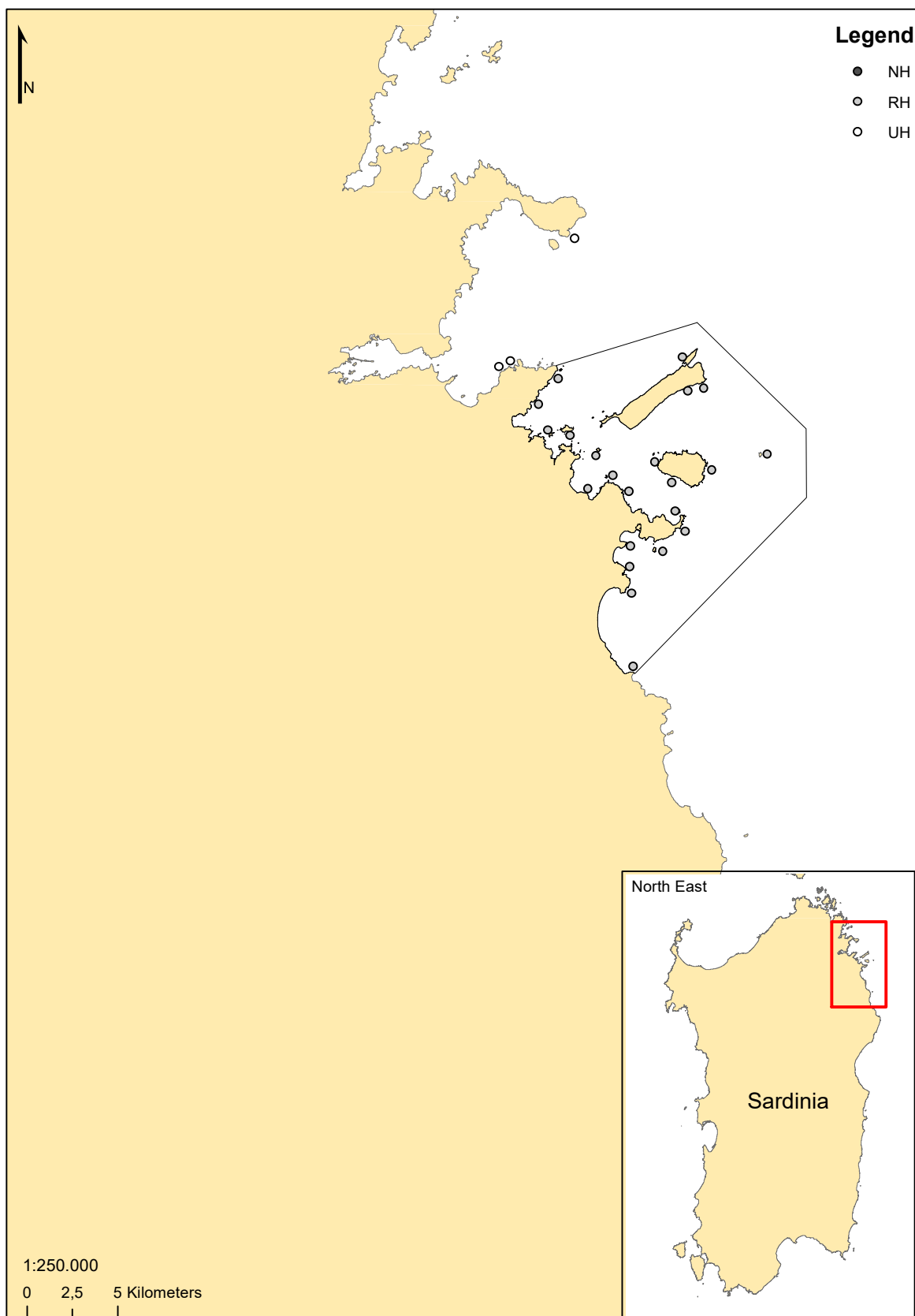

Fig. S4

Supplement: Supplemental Information 4 [file peerj-10-12971-s004.pdf]

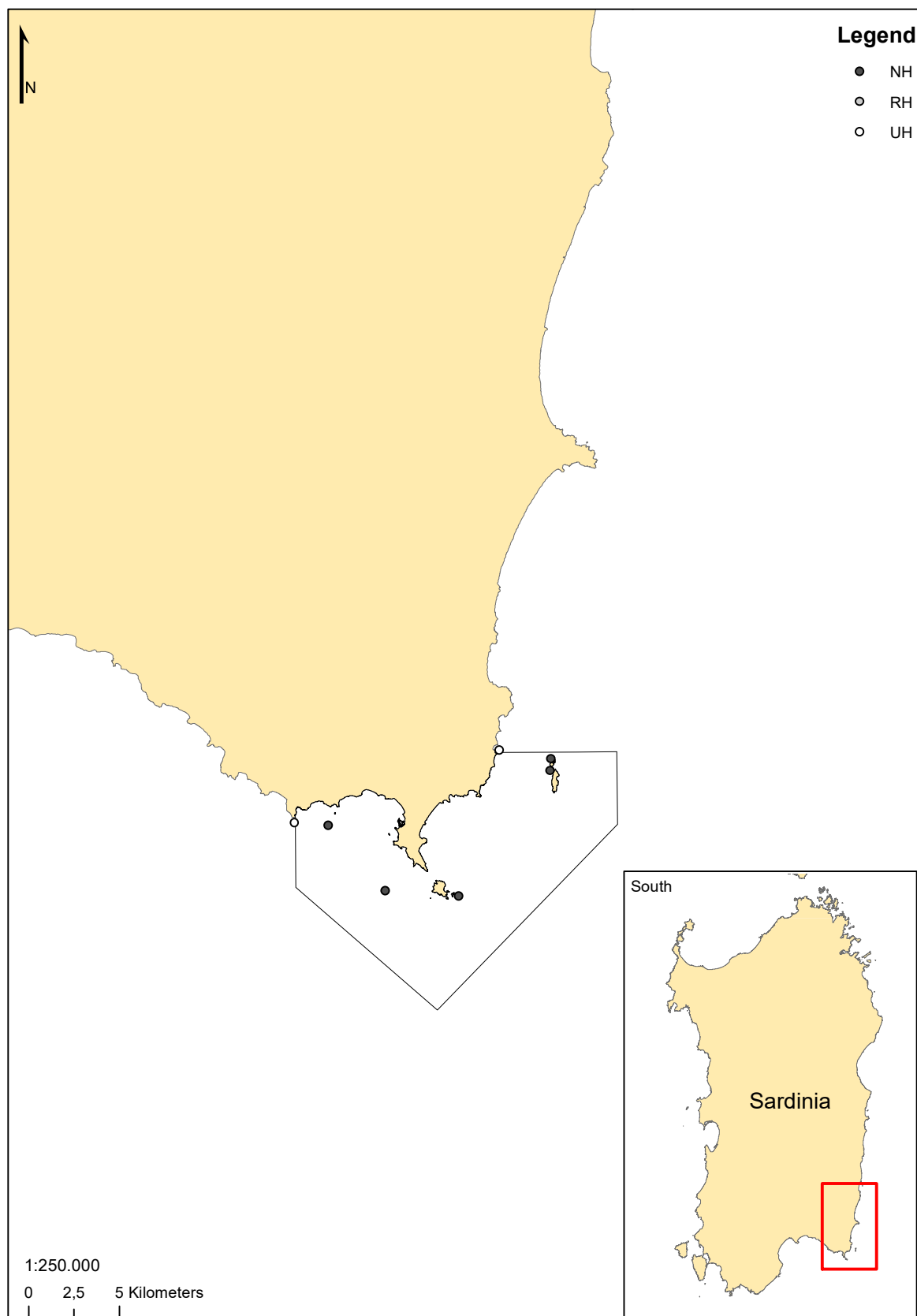

Fig. S5

Supplement: Supplemental Information 5 [file peerj-10-12971-s005.pdf]
